# Skyrmion Lattice Topological Hall Effect near Room Temperature

**DOI:** 10.1038/s41598-018-33560-2

**Published:** 2018-10-19

**Authors:** Maxime Leroux, Matthew J. Stolt, Song Jin, Douglas V. Pete, Charles Reichhardt, Boris Maiorov

**Affiliations:** 1 Materials Physics and Applications Division, Los Alamos National Laboratory, Los Alamos, New Mexico 87545 USA; 2 Department of Chemistry, University of Wisconsin-Madison, 1101 University Avenue, Madison, Wisconsin 53706 USA; 3 Centre for Integrated Nanotechnologies, Sandia National Laboratories, Albuquerque, New Mexico 87185 USA; 4 Theoretical Division, Los Alamos National Laboratory, Los Alamos, New Mexico 87545 USA

## Abstract

Magnetic skyrmions are stable nanosized spin structures that can be displaced at low electrical current densities. Because of these properties, they have been proposed as building blocks of future electronic devices with unprecedentedly high information density and low energy consumption. The electrical detection of an ordered skyrmion lattice via the Topological Hall Effect (THE) in a bulk crystal, has so far been demonstrated only at cryogenic temperatures in the MnSi family of compounds. Here, we report the observation of a skyrmion lattice Topological Hall Effect near room temperature (276 K) in a mesoscopic lamella carved from a bulk crystal of FeGe. This region coincides with the skyrmion lattice location revealed by neutron scattering. We provide clear evidence of a re-entrant helicoid magnetic phase adjacent to the skyrmion phase, and discuss the large THE amplitude (5 nΩ.cm) in view of the ordinary Hall Effect.

## Introduction

Magnetic skyrmions are nanometre size quasi-particles with a whirling spin configuration, which hold great potential as information carriers for electronic devices^1–5^. Magnetic skyrmions were first studied theoretically^6–8^, and have recently been evidenced experimentally in B20 chiral materials^9,10^ and in multilayer ferromagnetic thin films^11–13^. Most skyrmionic materials have in common^1,2^ the presence of an antisymmetric exchange interaction, known as Dzyaloshinskii–Moriya (DM) interaction^14,15^, favouring spin canting in materials that would otherwise be ferromagnetic with parallel aligned spins. The DM interaction occurs in materials with spin-orbit coupling and a structure lacking inversion symmetry, such as the non-centrosymmetric “B20 magnets” crystals: MnSi^9^ and FeGe^10^. In multilayer ferromagnetic thin films, the DM interaction is engineered by combining ultra thin layers of heavy metal and ferromagnetic materials, in which the heavy metal provides the large spin-orbit coupling while the interface between layers breaks the inversion symmetry^3^. For a large enough DM interaction, such as that found in B20 magnets, the ground state of the material is helical. In this state, the spins form helices with a pitch of a few tens of nanometres typically. This pitch scales as J/D, where D is the DM interaction constant and J is the exchange energy^1^. This pitch is referred to as the helical wavelength and it sets the skyrmion size in bulk materials. The topologically non-trivial whirling configuration of skyrmions spin texture can be characterised by the topological charge that is ±1 for an individual skyrmion, and null for topologically trivial structures such as magnetic bubbles. The topologically non-trivial structure of magnetic skyrmions makes them relatively stable because they cannot be continuously deformed to another magnetic state^1,16^.

A key advantage of magnetic skyrmions as stable nano-objects is that they can be displaced using relatively low electrical currents^3,17,18^. This low critical current for depinning stems from the relative insensitivity of skyrmions to disorder due to the strong Magnus force contribution to the skyrmion dynamics that allows skyrmions to bypass defects^1,4,5,19^. Such current manipulation enables not only data storage but also logic devices using magnetic “racetrack” type circuits^2,3^. Skyrmions nanometre size (10 nm ≡ 6 Tbit/in^2^), stability and low critical current (10^6^ A/m^2^) could thus yield non-volatile electronic devices with unprecedentedly high information density and low energy consumption.

One of the key issues for future “skyrmionic” devices is to develop a method for detecting skyrmions through electrical means. In multilayers thin films, this was achieved very recently at room temperature for isolated skyrmions and skyrmions in amorphous lattices^12,13,20,21^. Although the detection of skyrmions in multilayers was first attributed to the Topological Hall Effect (THE)^12^, it was later shown by Fert *et al*. to be caused by the Anomalous Hall Effect (AHE)^13^. This AHE originates from the step change in magnetisation introduced by skyrmions in a ferromagnetic background. By contrast, in macroscopic bulk crystals, the skyrmion THE has been detected and correlated with skyrmions imaging, but only in the MnSi family of compounds at cryogenic temperatures^18,22–24^. This THE originates from electrons accumulating a Berry phase as they travel through skyrmions spin configuration, which acts as an effective magnetic field (${B}_{eff}$).

To first order, the skyrmion THE amplitude is proportional to ${B}_{eff}$, which itself is inversely proportional to a skyrmion cross-section area. The skyrmion THE is usually a few n${\rm{\Omega }}$.cm^22,23,25^ (although claims of large skyrmions THE values up to 1000 n${\rm{\Omega }}$.cm have been reported^26–30^), and it arises in addition to the Ordinary Hall Effect (OHE) and AHE in the transverse resistivity of skyrmionic compounds. The detection of skyrmions via the AHE is also not readily applicable to bulk skyrmion compounds, because the skyrmion lattice does not introduce a significant change in magnetisation.

Skyrmions in bulk crystals are in fact quite different from skyrmions in multilayers. Multilayers host metastable Néel-type “pancake-like” skyrmions with extreme aspect ratio, which occur in individual ferromagnetic layers of the order of one nanometre thick while extending out laterally to tens or hundreds of nm, and they are either found isolated^11–13^ or in an amorphous lattice^12^. By contrast, skyrmions in bulk crystals are Bloch-type three-dimensional elongated “tubes” which form a thermodynamically stable triangular lattice with long-range order^9^. Thus, skyrmions in bulk crystals share similarities with other 3D elastic lines systems in physics, such as dislocations and superconducting vortices, whereas the physics of skyrmions in multilayers is more similar to that of domain walls and magnetic bubbles in magnetic thin films, with a strong influence of the sputtered films inherent disorder. For instance, electrical manipulation of skyrmions in multilayers^31–36^ still requires current densities comparable to those required to move domain walls in racetrack type memories^2,3^, which is orders of magnitude larger than the current densities required to move skyrmions in clean single crystals^17,18,37^.

Recently, two bulk crystalline materials have been shown by Lorentz Transmission Electron Microscopy (LTEM) and Small Angle Neutron Scattering (SANS) to have a skyrmions lattice near or at room temperature: Co_*x*_-Zn_*y*_-Mn_*z*_ alloys^38,39^, and stoichiometric cubic FeGe^10,37,40–42^. In FeGe, magnetisation^43^, specific heat measurements^44^, and microwave absorption spectroscopy^45^ also identified several magnetic phases compatible with skyrmions. Transport studies in FeGe thin films^26–30^ reported an extraordinarily large Hall signature (up to 1000 n${\rm{\Omega }}$.cm in 36 nm thin films) that occurs in an “extended” phase spanning from cryogenic temperatures to the Néel temperature. These studies claim that this Hall signature corresponds to a skyrmion THE as it cannot be explained by the AHE and, at first, it appeared to match the extended skyrmion phase observed in LTEM imaging of thin lamellae of FeGe bulk crystals^10,37,40^.

However, the nature of this Hall signature remains actively debated. In particular, it was noticed that the “extended” phase, in which the Hall signature is observed in thin films, spans a much larger range of the phase diagram than the extended skyrmion phase observed by LTEM in single crystals of comparable thickness^27^. In addition, Zhang *et al*.^46^ found no clear evidence for the presence of skyrmions in thin films of FeGe, and instead observed an extended helimagnetic phase. TEM cross-section imaging in FeGe thin films^30^ did observe skyrmions but only in the case that corresponds to applying the field in the plane of a 1000 nm-thick film. Very recent studies also found evidence for chiral bobbers coexisting together with skyrmions in FeGe crystals^47^ and thin films^30^. These chiral bobbers lies at the surface and only extend into the sample up to a few tens of nm. The large Hall signature in thin films of FeGe also differs from other known THE in bulk and multilayer skyrmion compounds. Qualitatively, the Hall signature is hysteretic, spanning from negative to positive field with some residual value at zero field, and no trace of the helical phase at low field is visible in the signal. Quantitatively, its value is difficult to account for within current theoretical models of skyrmion THE. Finally, a similar controversy occurred before for MnSi thin films in which there are skyrmions when the magnetic field is applied in-plane but not when it is applied perpendicular to the film^48–50^. Thus, there is still no consensus on skyrmion THE in FeGe thin films and no report of skyrmion THE in bulk FeGe crystals. This is somewhat surprising as the large THE value observed in thin films should make this a straightforward measurement in bulk crystals. However, the skyrmion THE is still a very nascent topic as there are very few confirmed skyrmion THE in the literature apart from the MnSi family of compounds. Thus there is only a very limited experimental data set to draw comparisons and to test the robustness of skyrmion THE theory.

Here, we demonstrate the first THE emerging from an ordered skyrmion lattice in a bulk crystal since the original skyrmion THE discovery in the MnSi family of compounds, and, what is more, near room temperature. Using high sensitivity Hall Effect measurements in FeGe crystals, we observe a skyrmion THE of amplitude +5 n${\rm{\Omega }}$.cm at 276 K (3°C). This THE coincides with the skyrmion lattice phase identified by small angle neutron scattering^42^, and its amplitude is as large as the THE observed in MnSi at 29 K. Adjacent to the skyrmion phase, we also observe the signature of a magnetic phase that is not present in the prototypical skyrmion compound MnSi, and merges into the inhomogeneous chiral spin state^51^. Finally, comparing THE in MnSi and FeGe we emphasise the quantitative and qualitative failings of the formula that is generally used to estimate the skyrmion THE.

## Results and Discussion

### Electrical Transport Measurements

**Figure 1:**
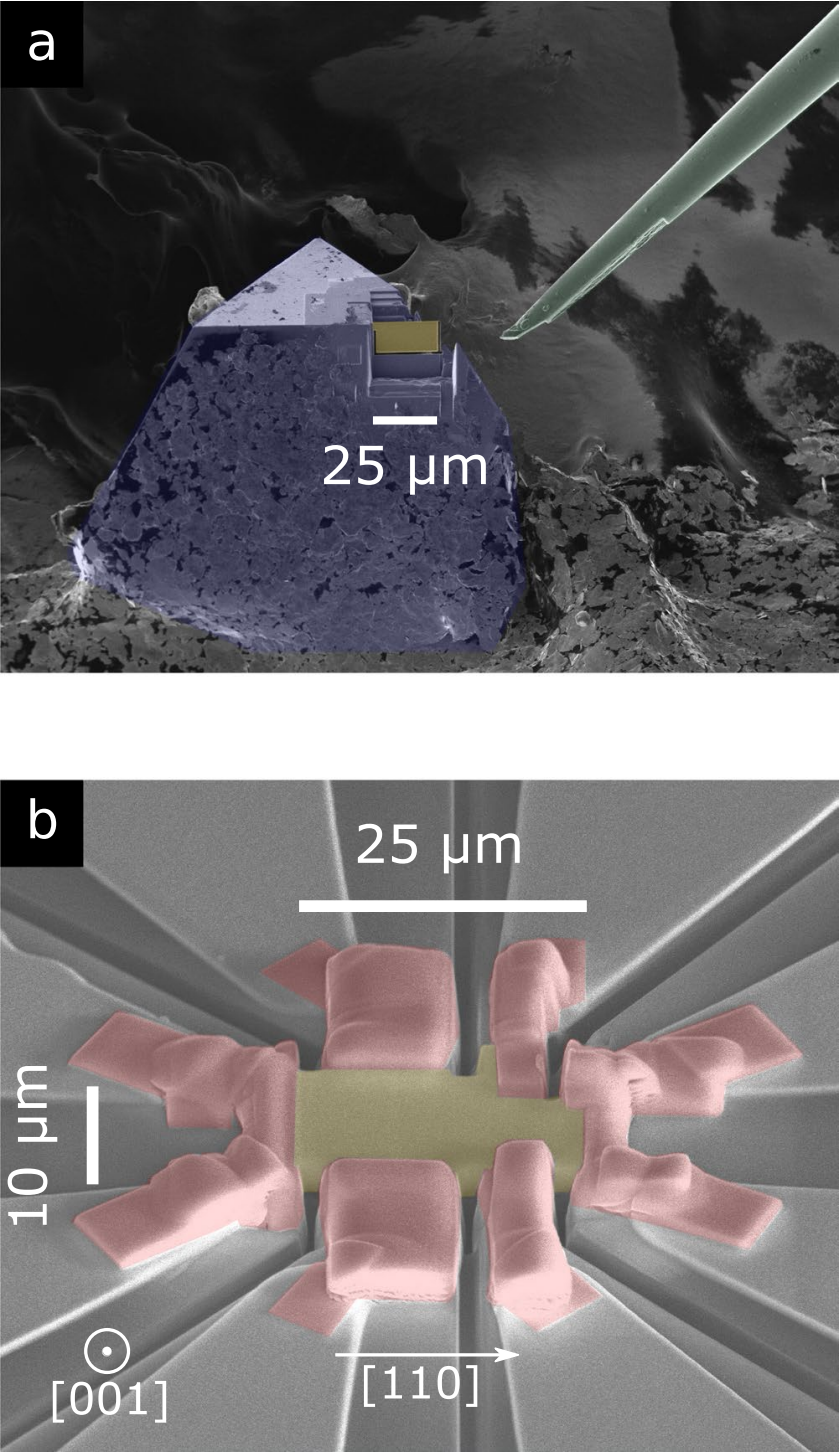
Nanofabrication of electrical transport sample by Focused Ion Beam (FIB). (**a**) Electron microscope picture. False colours: (*yellow*) lamella sample, (*blue*) FeGe single crystal, (*green*) FIB nanoprobe tip. FeGe lamella sample carved from a pyramidal FeGe single crystal, before final cut and lift-out with nanoprobe. The single crystal is electrically grounded to the FIB sample holder with silver paint. (**b**) Lamella with platinum electrodes on silicon oxide chip. Final dimensions: $10\times 25\times 0.75$ *μ*m^3^. The electrical current flows along [110] (see arrow) and the magnetic field is applied perpendicular to the flat face of the lamella along [001]. False colours: (*yellow*) lamella sample, (*red*) platinum contacts deposited by FIB, (*light grey*) evaporated platinum strips, (*dark grey*) silicon oxide chip.

**Figure 2:**
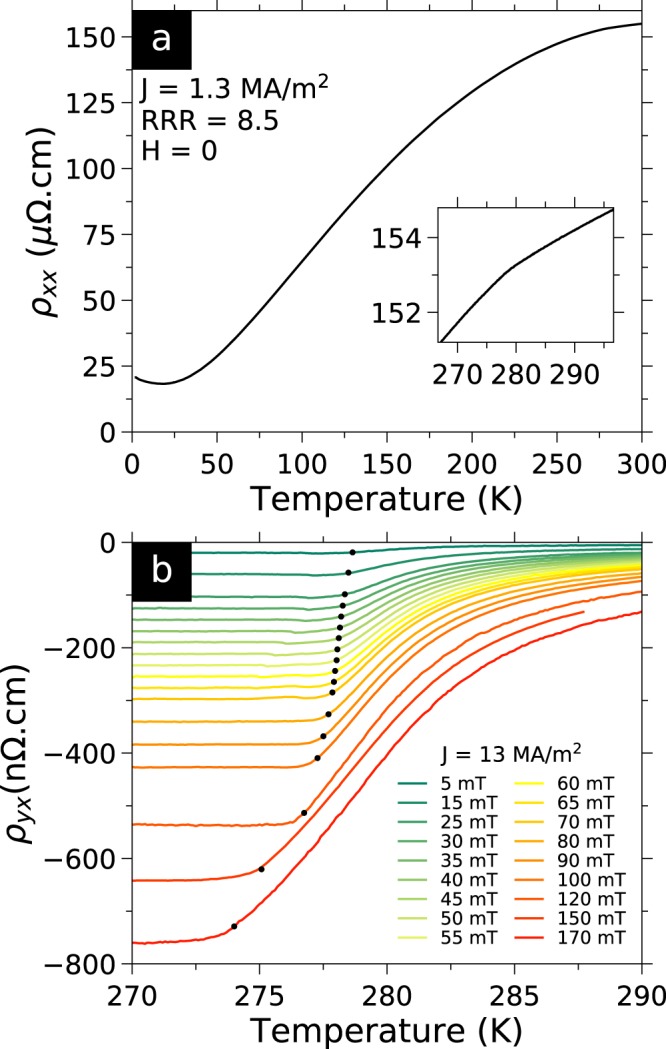
Electrical transport properties of the FeGe lamella. (**a**) Temperature dependence of the zero field longitudinal resistivity ${\rho }_{xx}$ of the sample. (Inset) Zoom on the kink around 279 K marking the magnetic transition. (**b**) Temperature dependence of the transverse resistivity ${\rho }_{yx}$ anti-symmetrised with respect to the magnetic field. The change in ${\rho }_{yx}$ marks the magnetic transition between the conical phase at low temperature and the paramagnetic/field polarised phases at high temperature. Black circles indicate the transition temperature defined using a 5% criterion and reported in Fig. 4.

We prepared a 0.75 *μ*m thick lamella of FeGe from a single crystal using a Focused Ion Beam microscope (FIB). Figure 1a shows the lamella before lift-out from the single crystal, and Fig. 1b shows the lamella with Pt electrodes deposited by FIB. We then measured the electrical transport properties of this lamella which shows transport properties consistent with bulk crystals (see Supplementary Information). Figure 2a shows the temperature dependence of the longitudinal resistivity ${\rho }_{xx}$ in zero magnetic field. The overall dependence and absolute value of ${\rho }_{xx}$ is in agreement with the literature^28^. The residual resistivity ratio is 8.5, higher than the value of 4–5 typically found in thin films^27,28^ but below the values of 13 to 25 found in large single crystals^52,53^. The magnetic transition from the paramagnetic phase to the helical phase appears as a subtle but clear kink around 279 K in the zero-field ${\rho }_{xx}$ data (inset of Fig. 2a).

In Fig. 2b, we show the temperature dependence of the Hall Effect ${\rho }_{yx}$ of the lamella (using the same sign convention as in refs^22,27,28^, that ${\rho }_{yx}$ is positive for electrons dominated transport). Electrical conduction appears to be predominantly hole type as ${\rho }_{yx}$ is negative around *T*_*N*_ (270–290 K). The onset of the increase in ${\rho }_{yx}$ with increasing temperature, marks the transition from the conical to the paramagnetic phase indicated by black circles in Fig. 2. All ${\rho }_{yx}(T)$ curves are constant in the conical phase in the temperature range we measured. For each magnetic field value, we define a transition temperature as 5% of the increase in ${\rho }_{yx}(T)$ between the flat baseline at 270 K and the value at 290 K. The magnetic field dependence of this transition temperature is in good agreement with previous magnetisation studies in a bulk sample^43,51^.

**Figure 3:**
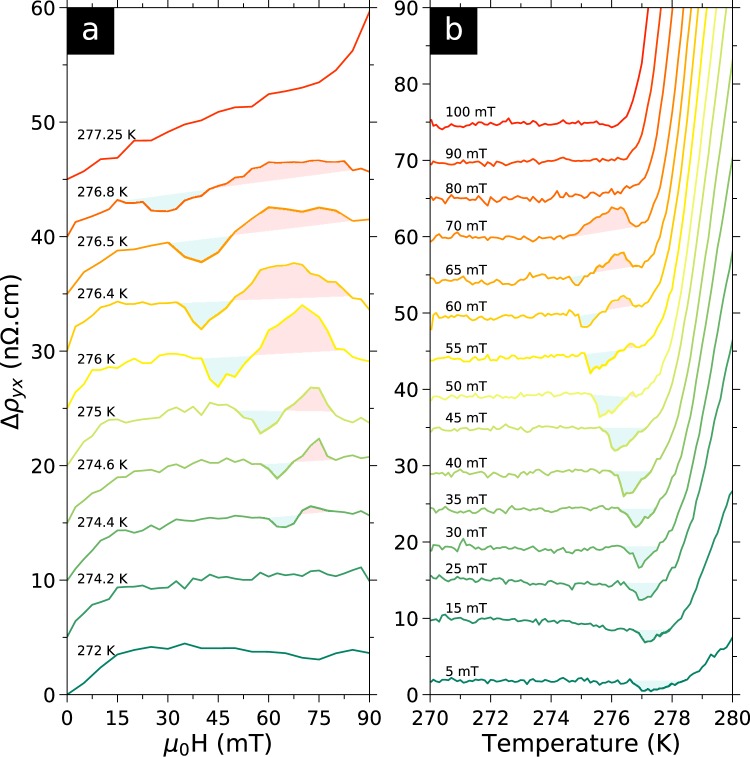
Temperature and field dependence of the deviation to the linear normal and anomalous Hall Effects. (**a**) Deviation ${\rm{\Delta }}{\rho }_{yx}$ (see text) as a function of applied magnetic field for several temperatures. Curves were shifted up for clarity. The helical phase occurs below the slope change at ≈15 mT, while the conical phase occurs above. The field polarised state appears as a rapid upturn at higher field (shown at 277.25 K). The blue shaded regions emphasise the local minimum which appears to emerge from the zero field magnetic transition (cf. Fig. 4). The origin of this minimum is still unidentified but might be a re-entrant ICS state. The red shaded regions emphasise the local maximum which coincides with the skyrmions lattice phase identified by SANS^42^ in bulk crystals (cf. Fig. 4). We attribute this maximum to the THE of skyrmions. (**b**) Deviation ${\rm{\Delta }}{\rho }_{yx}$ as a function of temperature for several applied magnetic fields. Curves were shifted up for clarity. The same features are observed.

The Hall Effect in skyrmionic compounds typically consists of three terms:

1 $${\rho }_{yx}={\rho }^{{\rm{OHE}}}+{\rho }^{{\rm{AHE}}}+{\rho }^{{\rm{THE}}}$$

the first term is the Ordinary Hall Effect ${\rho }^{{\rm{OHE}}}={R}_{0}{\mu }_{0}H$ proportional to the applied magnetic field, the second term is the Anomalous Hall Effect ${\rho }^{{\rm{AHE}}}={R}_{s}M$ proportional to the magnetisation *M* of the sample and the third term is the THE induced by the spin texture of skyrmions. Here, we find that ${\rho }_{yx}(H)$ curves are linear in the conical phase, as expected. Indeed the Ordinary Hall Effect and the Anomalous Hall Effect are both linear in *H*, as the magnetisation is proportional to *H* in this phase^43^. So, to better see variations from the conical state, we subtracted the total linear contribution from ${\rho }_{yx}(H)$ curves in the same manner as ref.^22^. The slope of the linear fit of ${\rho }_{yx}(H)$ in the conical phase (between 270 and 271K), is −4.3164 n${\rm{\Omega }}$.cm/mT with ${r}^{2}=0.99998$. We subtracted this slope from all ${\rho }_{yx}(H)$ curves and defined the resulting deviation of ${\rho }_{yx}(H)$ to the conical phase as ${\rm{\Delta }}{\rho }_{yx}$. ${\rm{\Delta }}{\rho }_{yx}(H)$ curves are shown in Fig. 3a (shifted for clarity). As the conical contribution is constant in temperature, converting ${\rho }_{yx}(T)$ curves to ${\rm{\Delta }}{\rho }_{yx}(T)$ corresponds to adding an offset to each curve shown in Fig. 2b.

### Signatures in the Hall Effect

In Fig. 3a, the ${\rm{\Delta }}{\rho }_{yx}(H)$ curves below 274.4 K show several features. A change in slope at ≈15 mT separates the helical and conical phases, at low and high field respectively. ${\rm{\Delta }}{\rho }_{yx}(H)$ curves are flat in the conical phase, consistent with the definition of ${\rm{\Delta }}{\rho }_{yx}$. The upturn at higher fields (shown at 277.25 K) corresponds to the field polarised state. The helical-to-conical signature disappears above ≈277 K, in agreement with magnetisation studies^43,51^. In addition to the helical, conical and field polarised states, we observe a local maximum and minimum that appear simultaneously inside the conical phase. The maximum and minimum have maximum amplitude at 276 K of ≈+5 n${\rm{\Omega }}$.cm and ≈−3 n${\rm{\Omega }}$.cm, respectively.

**Figure 4:**
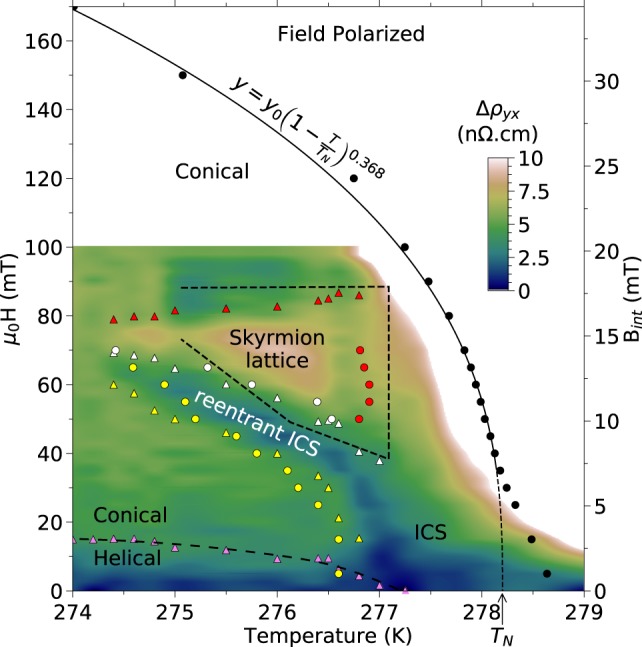
*H-T* diagram of the deviation to the linear Hall Effect. Deviation ${\rm{\Delta }}{\rho }_{yx}(T,H)$ (see text) deduced from ${\rho }_{yx}(H)$ and ${\rho }_{yx}(T)$ curves. (*Black circles*) transition separating the conical and field polarised phases, as defined by the 5% criterion in ${\rho }_{yx}(T)$ curves in Fig. 2b. The solid line is a fit to the high field part with critical exponent $\beta =0.368$ for 3D Heisenberg spins, as previously observed^51^. *T*_*N*_ extrapolates to 278.2 K, in agreement with previous studies^51^. (*Pink triangles*) low field change in slope in ${\rm{\Delta }}{\rho }_{yx}(H)$ curves, coinciding with the helical to conical transition. Dashed line is a guide to the eye. (*Yellow triangles and circles*) low field and low temperature onset of the local minimum in ${\rm{\Delta }}{\rho }_{yx}(H)$ and ${\rm{\Delta }}{\rho }_{yx}(T)$, respectively. (*White triangles and circles*) point of inversion between the minimum and maximum. (*Red triangles and circles*) high field and high temperature onset of the maximum. (*Dotted edge polygon*) Skyrmion lattice phase measured by SANS^42^ for *H*//[100] in a spherical bulk crystal after correcting for demagnetising effects. From this, we attribute the maximum in ${\rm{\Delta }}{\rho }_{yx}$ to the topological Hall Effect of the skyrmion lattice. No SANS data in the longitudinal geometry showing the six-fold scattering, is published at temperature below the open end of the polygon. The origin of the local minimum is still unidentified but it appears to continue into the inhomogeneous chiral spin state^51^ between *T*_*N*_ and the helical state.

In Fig. 3b, from 270 to ≈274.5–277 K, ${\rm{\Delta }}{\rho }_{yx}(T)$ is constant in temperature. This corresponds to the helical or conical states, at low or high magnetic fields respectively. Above ≈278 K there is a large upturn corresponding to the transition to the paramagnetic or field polarised states. Around 276 K, we also observe a local maximum and minimum. The minimum is present from 5 to 65 mT, whereas the maximum is present in a narrower field range from 55 to 70 mT. The maximum and minimum observed in ${\rm{\Delta }}{\rho }_{yx}(H)$ and ${\rm{\Delta }}{\rho }_{yx}(T)$ are consistent in both locations and amplitudes, and have never been reported before. To gain further insights on their origin we combined ${\rm{\Delta }}{\rho }_{yx}(H)$ and ${\rm{\Delta }}{\rho }_{yx}(T)$ data sets in Fig. 4 to produce a colour map (colour maps made from either data sets are shown in the Supplementary Information).

### Skyrmion Topological Hall Effect

Figure 4 shows that the local maximum occurs in a region of field and temperature, with a shape and location reminiscent of the skyrmion phase in MnSi. After accounting for demagnetising effects (right axis of the *H-T* diagram) following the process of ref.^54^ (see Supplementary Information), this region corresponds quantitatively in field and temperature to the region where a skyrmion lattice has been observed in bulk crystals of FeGe by SANS^42^. We therefore conclude that this local maximum is the Topological Hall Effect induced by skyrmions magnetic texture. We note that this is the first report of a skyrmion Topological Hall Effect near room temperature in a bulk crystal. However, as mentioned in introduction, claims of a skyrmion THE up to 200 times larger than our measurement and spanning from cryogenic temperature to near room temperature have been reported before in FeGe thin films^27–30^. We also note that in light of ref.^48^ where the authors show contributions of THE which are not due to the skyrmion magnetic phase but rather to the conical phase, we do not exclude the contribution (even very small) of other non-collinear spin configurations in our THE measurement.

The maximum amplitude of this THE is ≈+5 n${\rm{\Omega }}$.cm at 276 K, a value very similar to that of MnSi^22^ at 29 K. This is contrary to what is expected from the small ${B}_{eff}$ of FeGe produced by its larger skyrmions. However, ${B}_{eff}$ is not the only factor determining the THE amplitude. The exact amplitude of the THE depends on the details of the band structure, but the THE is usually approximated as^22,25,55^:

2 $${\rm{\Delta }}{\rho }_{yx}^{max}\approx P\mathrm{.}{B}_{eff}\mathrm{.}{R}_{0}$$

where *P* is the local spin-polarisation of the conduction electrons in the skyrmion state, ${B}_{eff}$ is the effective magnetic field and *R*_0_ is the ordinary Hall Effect constant. Here, we find (see methods) that in FeGe:

3 $${\rm{\Delta }}{\rho }_{yx}^{THE}=0.082\times -\,0.74\times -\,0.73\,{10}^{-8}\approx +\,45\,{\rm{n}}{\rm{\Omega }}{\rm{.}}\,{\rm{cm}}$$

And this value is of the same order as the approximate THE value in MnSi:

4 $${\rm{\Delta }}{\rho }_{yx}^{THE}\mathrm{=0.09}\times -\,13.15\times 0.12\,{10}^{-8}\approx -\,140\,{\rm{n}}{\rm{\Omega }}.\,{\rm{cm}}$$

We note that *P* is similar in both compounds, ${B}_{eff}$ is 18 times smaller in FeGe than in MnSi, and *R*_0_ is 6 times larger in FeGe than in MnSi. Overall, the THE amplitude estimated via Eq. 2 is thus only three times smaller in FeGe than in MnSi.

Both THE values clearly overestimate the experimental values of the THE (≈+5 n${\rm{\Omega }}$.cm in both MnSi and FeGe). This overestimation has been known for some time in MnSi and attributed to the factor *R*_0_^25^. Recently, it was pointed out that this estimate is only an upper bound of skyrmions THE in multilayers thin films^13^ as skyrmions THE only occur in the ferromagnetic layers and the short mean free path in thin films could also reduce the THE. We also point out that the sign of the THE calculated using Eq. 2 appears to be opposite to what is measured in MnSi^22^. In addition, as mentioned above, bulk crystals and thin films of FeGe are very different in terms of skyrmions phase extent and THE, which may point toward fundamental skyrmions physics or extrinsic contributions to the skyrmion THE. For instance, recent theoretical efforts^56^ show that a range of sign and amplitude is to be expected for the THE depending on coupling strength and electronic scattering rates. Looking beyond this ongoing debate, we note that at least the THE estimates for MnSi and FeGe are of the same order of magnitude in absolute value: 140 n${\rm{\Omega }}$.cm for MnSi and 45 n${\rm{\Omega }}$.cm for FeGe. Thus, this could explain why the experimental values of the THE are so similar in both compounds.

We also emphasise that the larger *R*_0_ of FeGe appears to compensate the smaller ${B}_{eff}$. This means that the charge carrier density, and more generally the band structure, is a tool as important as skyrmions effective magnetic field in achieving measurable THE. For instance, in Pt/Co/Ir multilayer thin films, the skyrmion THE was estimated to be immeasurably small: ${\rm{\Delta }}{\rho }_{yx}^{THE}=0.0017\,{\rm{n}}{\rm{\Omega }}.\,{\rm{cm}}$ as ${R}_{0}={2.10}^{-11}\,{\rm{\Omega }}$.m.T^−1^ ^13^. This value is more than 300 times smaller than the value $|{R}_{0}|={\mathrm{7.3.10}}^{-9}\,{\rm{\Omega }}$.m.T^−1^ in FeGe, which suggest that FeGe is a compound of choice for the study of the THE near room temperature.

### Signature of a Re-entrant Helicoid Magnetic Phase

The local minimum observed in ${\rm{\Delta }}{\rho }_{yx}$ in Fig. 4, is new to all compounds with clear signatures of vortex-like cylindrical skyrmions. A positive THE has been observed in MnSi^22^, and a negative THE was found in compounds such as Mn_1−*x*_Fe_*x*_Si^23^, but a positive and negative THE have never been observed consecutively, up to our knowledge.

Polycrystalline MnGe does show adjacent maximum and minimum in ${\rho }_{yx}$^57^, but the amplitude of this effect is 50 times larger (≈200 n${\rm{\Omega }}$.cm) and spans over 10 Tesla and 100 Kelvin. Thus, this is very different from the phenomenon we observe in FeGe. In addition, the accepted interpretation of the behaviour of MnGe does not involve *cylindrical* skyrmions but a spin structure periodic in all three dimensions undergoing a topological phase transition through the pair annihilation of hedgehogs and anti-hedgehogs topological spin singularities^57^.

In the ${\rm{\Delta }}{\rho }_{yx}(H)$ curves of Fig. 3a and the *H-T* diagram of Fig. 4, the local minimum tracks and ends with the lowest temperature of the skyrmions phase. But at higher temperatures it continues into the inhomogeneous chiral spin state^51^ (ICS). Thus, the local minimum delimits a region which appears to compete with the skyrmion lattice phase but shares the signature of the helical and ICS states. Theoretical calculations^43^ show that the hierarchy of close energy scales yields several possible magnetic phases in the vicinity of *T*_*N*_, including: +*π* skyrmions, −*π* skyrmions, half-skyrmions squares lattice and re-entrant helicoid. The position of the local minimum in the phase diagram, bordering the skyrmion lattice phase, and its sign opposite to the THE of the skyrmion lattice, suggest +*π* skyrmions with a positive effective magnetic field and negative THE; however, SANS does not show an other ordered skyrmion region^42^. Thus, this suggests that the local minimum corresponds to a re-entrant ICS or helicoid state. Such re-entrant phase has never been observed before in skyrmion lattice systems, up to our knowledge. It also underscores the fact that, although magnetic and transport measurements show features^43,51^ at similar locations, the latter indicate a very different origin for the re-entrant phase.

### High Current Density Measurements of Hall Signatures

Only two studies have ever reported a change in THE signal as a function of current density, and both are in MnSi^18,24^. When increasing the current density, the studies observed that: at first skyrmions are pinned and the THE signal is constant; then above a critical current density ${J}_{c}$ skyrmions start to move and the THE signal decreases; finally at even higher current density skyrmions are in a flow regime and the THE signal is constant again. The reduction in skyrmion THE amplitude at the transition from a pinned to a flow regime is explained by the emerging electric field induced by moving skyrmions^18^. The first study in thin single crystals of MnSi^18^ found a decrease in THE from +5 n${\rm{\Omega }}$.cm to +2 n${\rm{\Omega }}$.cm after skyrmions start to move at a critical current density of ${J}_{c}\approx 0.5$ MA/m^2^. The second study showed that ${J}_{c}$ values are sample dependent as measurements on two different MnSi nanowires^24^ found ${J}_{c}\sim 27$ and 72 MA/m^2^. Ultrasound experiments on thicker (mm-sized) single crystals of MnSi report even lower ${J}_{c}$ values of ~50 kA/m^2^ ^58^.

**Figure 5:**
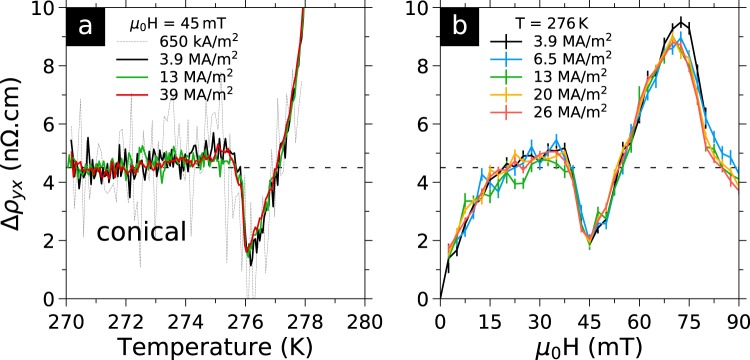
Deviation to the linear Hall Effect for several current densities. (**a**) ${\rm{\Delta }}{\rho }_{yx}(T)$ curves (see text) at 45 mT. Only the local minimum is present. No significant changes occur for current densities between 0.65 and 39 MA/m^2^, evidencing the absence of self-heating effect. Close inspection of raw data reveals only 10 mK of heating from 3.9 to 39 MA/m^2^. (**b**) ${\rm{\Delta }}{\rho }_{yx}(H)$ curves at 276 K. Error bars are +/− the estimator of the standard error of the mean. No changes occur for current densities between 6.5 and 26 MA/m^2^ in either the slope change at ≈15 mT marking the helical to conical phase transition, the local minimum at 40 mT, and the local maximum at 75 mT which we attribute to the THE of the skyrmion lattice phase. Data at 3.9 MA/m^2^ might show a 0.5 n${\rm{\Omega }}$.cm increase of the maximum at 75 mT, which would be compatible with slower or pinned skyrmions, but this is very close to the noise level.

In Fig. 5 we show ${\rm{\Delta }}{\rho }_{yx}(T)$ for different current densities. At 45 mT, in Fig. 5a, we show results outside the skyrmion area to test for Joule heating from the contacts or sample. At this field only the local minimum is present and no significant changes occur either in the position or amplitude of the minimum for current densities between 0.65 and 39 MA/m^2^. Close inspection of the data reveals a small 10 mK heating at 39 MA/m^2^ with respect to 3.9 MA/m^2^, indicating the absence of any significant heating. In Fig. 5b, we show ${\rm{\Delta }}{\rho }_{yx}$ at 276 K for current densities ranging from 6.5 to 26 MA/m^2^. At this temperature, the field sweep encompasses several phases. Starting at low fields, one can see the change of curvature in ${\rm{\Delta }}{\rho }_{yx}(H)$ corresponding to the helical phase below 15 mT, as well as the local minimum at 40 mT, and the THE caused by skyrmions at 75 mT. We observe no significant changes in neither of these signatures. Difference between the data obtained at 3.9 MA/m^2^ with the data taken at other currents could indicate a 0.5 n${\rm{\Omega }}$.cm increase of the THE at 75 mT, nevertheless this difference is barely above noise level, so it must be taken only as indication. Our experimental resolution of ≈0.8 nV/$\sqrt{{\rm{Hz}}}$ is insufficient to measure the THE below ≈1 MA/m^2^ as in such a small sample (10 *μ*m width) it translates to 0.1 nV.

As we do not observe any change in the THE signal as a function of current density, we cannot conclude whether skyrmions are in the pinned or flow regime. However, direct TEM observation of skyrmion motion in a 100 nm thick FeGe lamella found a critical current for skyrmion depinning of ${J}_{c}\sim 50$ kA/m^2^ at $T=270$ K^37^. We therefore suggest that the current densities of our experiment place skyrmions in a flow regime and that a hypothetical pinned regime may exist at lower current density. In that case, FeGe would have a larger THE than that of MnSi in the skyrmion flow regime (≈+2 n${\rm{\Omega }}$.cm). Also, by analogy with MnSi, the THE of +5 n${\rm{\Omega }}$.cm at 13 MA/m^2^ that we measure in FeGe could maybe increase by a factor of two in the hypothetical pinned regime.

## Conclusions

We report the first observation of a skyrmion THE emerging from an ordered skyrmion lattice in a bulk crystal since the original skyrmion THE discovery in the MnSi family of compounds. Using high resolution Hall Effect measurements in a mesoscopic FIB lamella extracted from a single crystal of FeGe, we observe a 5 n${\rm{\Omega }}$.cm THE in a region of field and temperature close to *T*_*N*_ where a skyrmion lattice has been observed in bulk samples using SANS^42^. We also report the signature of a re-entrant magnetic phase adjacent to the skyrmion phase and connected to the helicoid or inhomogeneous chiral spin state. We argue that the large THE amplitude indicates that the smaller effective magnetic field produced by the bigger FeGe skyrmions is compensated by a larger ordinary Hall constant. The near-room temperature (276 K) and lower magnetic fields (~70 mT) at which it occurs, as well as its sign and amplitude, make this discovery highly relevant for theoretical and technological reasons. Technological, as the detection of a near-room temperature skyrmion THE in a clean single crystal establishes FeGe as a promising compound for applications, whether in bulk crystal or thin film form. Theoretical, as the current skyrmion THE model is only a “qualitative” predictor of its amplitude (and, more problematically, its sign) and our experimental results provide a much-needed new reference for studying the skyrmion THE in clean single crystals. In addition, our skyrmion THE value in bulk FeGe crystals is similar to that in MnSi crystals, but it is up to 200 times lower than the maximum THE value claimed in FeGe thin films, which could point toward fundamental skyrmions physics or extrinsic contributions to the THE.

## Methods

### Crystal Growth

Cubic FeGe is a chiral stoichiometric binary B20 compound, crystallising in the non-centrosymmetric $P{2}_{1}3$ cubic space group, isostructural to MnSi. Small single crystals (≈100 *μ*m wide) were grown by chemical vapour transport as described in ref.^59^. These samples were also used in refs^41,45^.

### Nanofabrication

A lamella 0.75 *μ*m thick, 10 *μ*m wide and 25 *μ*m long, was carved out of a pyramidal single crystal, using a FEI Helios 600 FIB/SEM. This lamella is shown in Fig. 2a. We used the known crystallographic orientation of pyramidal single crystals of FeGe, namely the [111] triangular facets with [110] edges, to align the cuts so that the lamella is perpendicular to the [100] axis and its edges are along [110] axes. The lamella was then laid flat on a custom chip, and electrical contacts were made using standard FIB platinum deposition. 2-wire contact resistances ranged from 33 ${\rm{\Omega }}$ for the current contacts, to 40 and 48 ${\rm{\Omega }}$ for voltage contacts, a significant part of which comes from the intrinsically high resistivity of the FIB deposited platinum (1–2 m${\rm{\Omega }}$.cm per the manufacturer). The result is a lamella for which the magnetic field is applied along [100] and the electrical current is flowing along [110].

### Electrical Transport Measurements

We measured the longitudinal resistivity ${\rho }_{xx}$ and the transverse or Hall resistivity ${\rho }_{yx}$ in a Quantum Design PPMS with a 9 T vertical magnet, using a standard 4-wire procedure with a Linear-Research LR-700 AC resistance bridge functioning at a fixed frequency of 15.9 Hz. We used the same sign convention as in refs^22,27,28^, that ${\rho }_{yx}$ is positive for electrons dominated transport. This combination of sample and setup achieved a noise level of ≈0.8 nV/$\sqrt{{\rm{Hz}}}$, which is close to the resolution limit of the instrument (≈0.6 nV/$\sqrt{{\rm{Hz}}}$) and the intrinsic Johnson-Nyquist noise of the resistive platinum contacts (≈0.7 nV/$\sqrt{{\rm{Hz}}}$). Considering measurement duration and current density (>1 MA/m^2^), this translates into a noise level of typically less than 0.5 n${\rm{\Omega }}$.cm for the Hall resistivity ${\rho }_{yx}$, and 15 n${\rm{\Omega }}$.cm for ${\rho }_{xx}$, on a sample that is only $0.75\times 10\times 25$ *μ*m^3^ in size.

The temperature dependence curves, ${\rho }_{yx}(T)$, were measured by ramping up the temperature from 270 to 290 K for several applied magnetic fields, following a zero field cooled (ZFC) procedure. We used the standard magnetic field anti-symmetrisation procedure to remove the experimental offset. The offset measured is equivalent to a misalignment of the contacts of ≈3 *μ*m. ${\rho }_{yx}(H)$ curves were measured by cooling in zero field to the target temperature, raising the magnetic field, and then measuring ${\rho }_{yx}$ while stepping down the field to the opposite value. The field anti-symmetrisation procedure was again used to remove the experimental offset. A complete hysteresis cycle was also measured at 276 K (see Supplementary Information); compared to the ${\rho }_{yx}(H)$ curves, the only change in the initial ZFC ramp is an increase (from 15 to 30 mT) in the upper bound of the helical phase. An extension of the helical phase in magnetic field, has been observed in ZFC magnetisation measurements in Fe and Co doped MnSi^60^.

### Factors in the THE estimate

The polarisation factor is $P={m}_{s}/{\mu }_{sat}$, where *m*_*s*_ is the ordered magnetic moment (spontaneous magnetisation) in the skyrmion phase inferred from a linear extrapolation of the high-field data to *H* = 0 in an Arrott plot, and ${\mu }_{sat}$ is the saturated moment deduced from the Curie-Weiss law in the paramagnetic state above *T*_*N*_^22^. Ref.^28^ indicates that ${\mu }_{sat}=2.8\pm 0.1\,{\mu }_{B}/\mathrm{Fe}$ while ref.^51^ finds that ${m}_{s}\approx 10\,{\rm{emu}}/{\rm{g}}\mathrm{=0.23}\,{\mu }_{B}/{\rm{Fe}}$ at 276 K. We thus estimate the polarisation factor in FeGe to be $P\approx 0.23/2.8=0.082$, very similar to the value 0.09 in MnSi^22,25^. The effective magnetic field ${B}_{eff}$ induced by skyrmions magnetic texture is^22,25^:

5 $${B}_{eff}=-\,\frac{h}{e}(\frac{\sqrt{3}}{2{\lambda }_{S}^{2}})$$

As the helical period of FeGe is ${\lambda }_{S}=69.8$ nm just below *T*_*N*_^61^, the corresponding effective magnetic field is ${B}_{eff}=-\,0.74$ T, which is 18 times smaller than ${B}_{eff}=-\,13.15$ T in MnSi^25^ (${\lambda }_{S}=16.5$ nm). Comparing *R*_0_ values just above *T*_*N*_, we find *R*_0_ = +0.11 *μ*Ω.cm/T in MnSi at 35 K^22^ and *R*_0_ = −0.73 *μ*Ω.cm/T in our sample of FeGe at 290 K. Our value of *R*_0_ is very close to the value *R*_0_ = −1.09 *μ*Ω.cm/T in thin films of FeGe^28^. *R*_0_ is thus at least 6 times greater in FeGe than in MnSi.

## Electronic supplementary material

Skyrmion Lattice Topological Hall Effect near Room Temperature
