# Supplementary material for: Skyrmion Lattice Topological Hall Effect near Room Temperature
Source: Sci Rep. 2018 Oct 19;8:15510. doi: 10.1038/s41598-018-33560-2 (PMC6195581; doi:10.1038/s41598-018-33560-2)
Supplement: Supplementary file 1 — Skyrmion Lattice Topological Hall Effect near Room Temperature [file 41598_2018_33560_MOESM1_ESM.pdf]

Skyrmion Lattice Topological Hall Effect near Room Temperature

Maxime Leroux,[1] Matthew J. Stolt,[2] Song Jin,[2] Douglas V. Pete,[3] Charles Reichhardt,[4] and Boris Maiorov[1]

[1]*Materials Physics and Applications division, Los Alamos National Laboratory, Los Alamos, New Mexico 87545, United States*

[2]*Department of Chemistry, University of Wisconsin-Madison, 1101 University Avenue, Madison, Wisconsin 53706, USA*

[3]*Center for Integrated Nanotechnologies, Sandia National Laboratories, Albuquerque, New Mexico 87185, United States*

[4]*Theoretical Division, Los Alamos National Laboratory, Los Alamos, New Mexico 87545, United States*

Abstract

In this supplementary information we discuss sample thickness and bulk behavior. We show colormaps made either from the $\rho_{yx}(T)$ curves only (Fig. 1), or from the $\rho_{yx}(H)$ curves only (Fig. 2). We also show the full hysteresis cycle measured at 276 K (Fig. 3), and finally present the detailed calculations of the demagnetizing factor.

I. SAMPLE THICKNESS AND BULK BEHAVIOR

Although the lamella sample is thin, several experimental results indicate that it displays bulk behavior. At 750 nm thick, our sample is ≈ 800 times thinner than that of Ref. [1, 2], but is still more than 10 times thicker than the helical wavelength[3] $\lambda_S = 69.8$ nm. According to Lorentz TEM[4], a factor of 10 is in the bulk limit in terms of skyrmions phase extension. Additionally, the conical to field polarized transition line is in good agreement with the transition line measured in a bulk spherical sample in Ref. [1, 2], after correcting for demagnetizing effects (B_{int} right axis of Fig. 4 of the main text). This transition is defined in Fig. 2.b of the main text and reported as black circles in Fig. 4 of the main text. The high field dependence is also in excellent agreement with the exponent $\beta = 0.368$ for 3D-Heisenberg spins, as measured in a previous scaling study of FeGe[2]. Finally, the extrapolated value $T_N = 278.2$ K, is very close to $T_N = 278.6$ K found in Ref. [2]. As our skyrmion phase extension and the conical to field polarized transition line are both in good quantitative agreement with SANS and magnetization data, we thus observe a H-T phase diagram that is in excellent agreement with that of bulk samples.

II. HT DIAGRAMS

In Fig. 1 and Fig. 2 we show colormaps made, respectively, either from the $\rho_{yx}(T)$ curves only, or from the $\rho_{yx}(H)$ curves only. We recall the legend for the HT diagrams of Fig. 1 and 2. *(Black circles)* transition separating the conical and field polarized phases, as defined by the 5% criterion in $\rho_{yx}(T)$ curves in Fig. 2.b in the main article. The solid line is a fit to the high field part with critical exponent $\beta = 0.368$ for 3D Heisenberg spins, as previously observed[2]. T_N extrapolates to 278.2 K, also inline with the literature[2]. *(Pink triangles)* low field change in slope in $\Delta\rho_{yx}(H)$ curves, coinciding with the helical to conical transition. *(Yellow triangles and circles)* left onset of the local minimum in $\Delta\rho_{yx}(H)$ and $\Delta\rho_{yx}(T)$, respectively. *(White triangles and circles)* point of inversion between the minimum and maximum. *(Red triangles and circles)* right onset of the maximum. *(Dashed edge polygon)* Skyrmion lattice phase measured by SANS[5] for H//[100] in a spherical bulk crystal after correcting for demagnetizing effects. From this, we attribute the maximum in $\Delta\rho_{yx}$ to the THE of the skyrmion lattice. No SANS data in the longitudinal geometry showing the six-fold scattering, is published at temperature below the open end of the polygon. The origin of the local minimum is still unidentified but it appears to continue into the inhomogeneous chiral spin state[2] between T_N and the helical state. Dashed lines are guides to the eye.

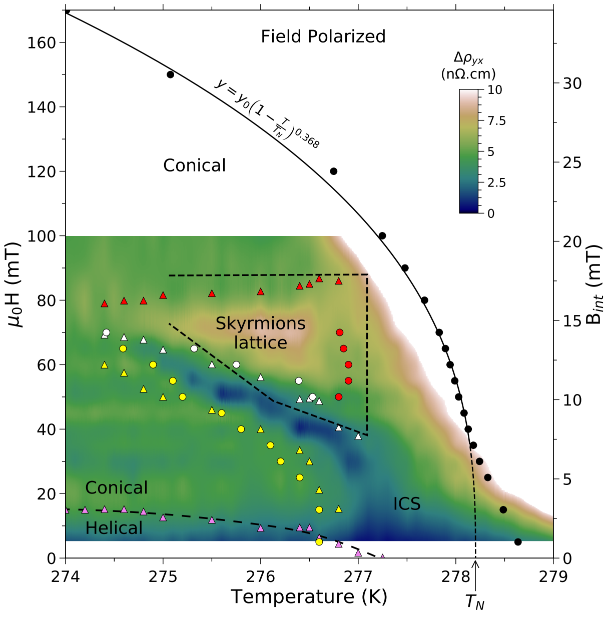

FIG. 1. **HT diagram of the deviation to the linear Hall effect from $\rho_{yx}(T)$ curves only.**

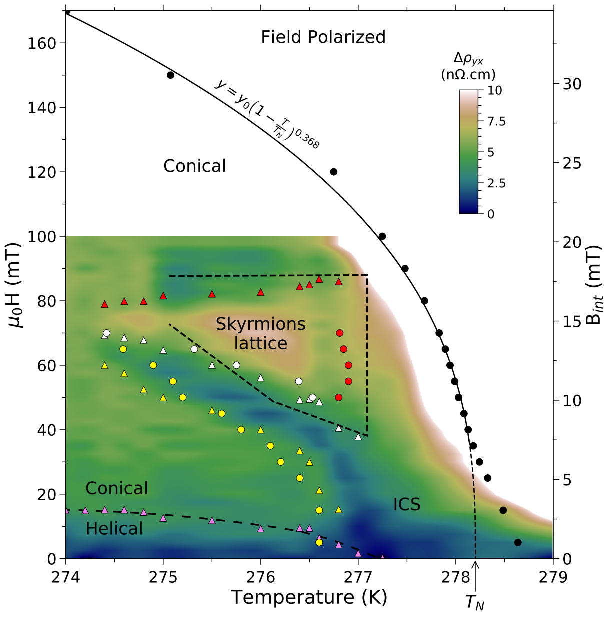

FIG. 2. **HT diagram of the deviation to the linear Hall effect from $\rho_{yx}(H)$ curves only.**

III. HYSTERESIS CYCLE

A complete hysteresis cycle of $\rho_{yx}(H)$ was measured at 276 K. We subtracted the slope -4.3164 nΩ.cm/mT from the $\rho_{yx}(H)$ curves measured during the hysteresis cycle, following the procedure explained in the main text (this slope corresponds to the 270-271 K average slope of $\rho_{yx}(H)$ in the conical state). The

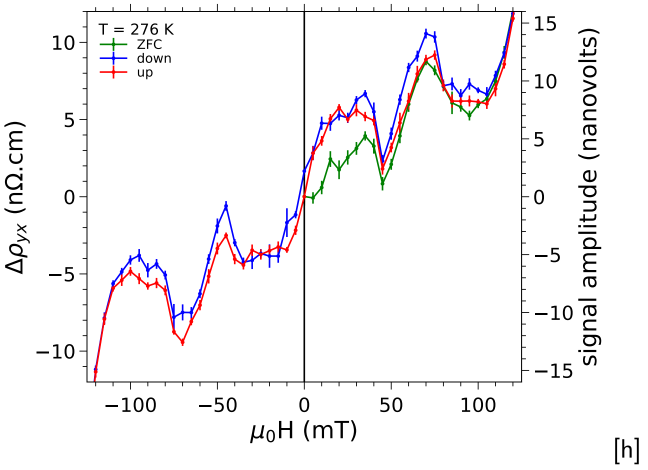

FIG. 3. **Deviation $\Delta\rho_{yx}(H)$ at 276 K for a complete hysteresis cycle.**

resulting curves are shown in Fig. 3. Compared to the $\rho_{yx}(H)$ curves, the only change in the initial ZFC ramp is an increase (from ≈ 15 to ≈ 30 mT) in the upper bound of the helical phase. An extension of the helical phase in magnetic field, has also been observed in ZFC magnetization measurements in Fe and Co doped MnSi[6].

IV. DEMAGNETIZING FACTOR

To calculate the demagnetizing factors we follow the procedure detailed in Ref. 7. The internal field is defined as

$$B_{int} = B_{ext} - \mu_0 N M \tag{1}$$

where $B_{ext} = \mu_0 H$, N is the demagnetizing factor and M is the magnetization. In the conical state of FeGe, M is linear with H to a good approximation[1, 2]. Stated otherwise, the magnetic susceptibility χ, defined as $M = \chi H$, is a constant, hence

$$B_{int} = \mu_0 H(1 - N\chi) \tag{2}$$

But χ is geometry dependent. After determining χ^{sph} from magnetization measurement on a sphere, χ^L for the lamella sample can be deduced from Eqn. (6.39) in Ref. 7:

$$\chi^L = \frac{\chi^{sph}}{1 - \chi^{sph}(N_{sph} - N_L)} \tag{3}$$

where N_{sph} and N_L are, respectively, the demagnetizing factors of the sphere and the lamella sample.

To determine χ^{sph}, we use the magnetization value $M = 0.20\mu_B$/Fe for $\mu_0 H = 0.05\,\mathrm{T}$ measured in a spherical sample and reported in Fig. 4 of Ref. 2 (M is constant in the conical state, in the

measured range of temperatures from 265 to 280 K). We also use: the unit cell volume from Ref. 8, $V_{u.c.} = 103.82\ 10^{-30}$ m^3, the fact that there are 4 formula units of FeGe per unit cell, and $\mu_B = 9.274\ 10^{-24}$ J/T. This yields

$$\chi^{sph} = \frac{dM}{dH} = \frac{\frac{0.20*\mu_B}{\frac{V_{u.c.}}{4}}}{\frac{0.05}{\mu_0}} \approx 1.80 \text{ (SI unit)} \tag{4}$$

Note that the value 1.80 for χ^{sph} that we deduced here from DC magnetization measurements is also in excellent agreement with the AC susceptibility measurements of Ref. [1] $\chi_{AC} = 22\,$emu/mol for $\mu_0 H_{AC} = 1\,$mT, yielding $\chi^{sph}_{AC} = 1.77$ in SI unit.

For a spherical FeGe sample such as the ones used in SANS[5] and magnetization measurements[1, 2], $N_{sph} = 1/3$ (see for instance Ref. [9] but note that it is in cgs units so that it finds $N = 4\pi \times 1/3$, which is 1/3 in SI units). Hence for the spherical sample:

$$B_{int} = \mu_0 H(1 - N_{sph}\chi^{sph}) = \mu_0 H\left(1 - \frac{1.8}{3}\right) = 0.4\,\mu_0 H \tag{5}$$

For our lamella sample, N is calculated from the dimensions of the sample using the analytical formula in Ref. [10] for a ferromagnetic prism with arbitrary dimensions a, b and c :

$$\begin{aligned} N = \frac{1}{\pi}\Bigg(& \frac{a}{2c}\log\left(\frac{b+\sqrt{a^2+b^2}}{-b+\sqrt{a^2+b^2}}\right) + \frac{b}{2c}\log\left(\frac{a+\sqrt{a^2+b^2}}{-a+\sqrt{a^2+b^2}}\right) \\ & + 2\,\mathrm{atan}\left(\frac{ab}{c\sqrt{a^2+b^2+c^2}}\right) + \frac{c}{2b}\log\left(\frac{-a+\sqrt{a^2+c^2}}{a+\sqrt{a^2+c^2}}\right) \\ & + \frac{1}{2bc}\left(b^2-c^2\right)\log\left(\frac{-a+\sqrt{a^2+b^2+c^2}}{a+\sqrt{a^2+b^2+c^2}}\right) + \frac{c}{2a}\log\left(\frac{-b+\sqrt{b^2+c^2}}{b+\sqrt{b^2+c^2}}\right) \\ & + \frac{1}{2ac}\left(a^2-c^2\right)\log\left(\frac{-b+\sqrt{a^2+b^2+c^2}}{b+\sqrt{a^2+b^2+c^2}}\right) + \frac{c}{ab}\left(\sqrt{a^2+c^2}+\sqrt{b^2+c^2}\right) \\ & + \frac{\sqrt{a^2+b^2+c^2}}{3abc}\left(a^2+b^2-2c^2\right) + \frac{a^3+b^3-2c^3}{3abc} \\ & + \frac{1}{3abc}\left(-\left(a^2+b^2\right)^{1.5} - \left(a^2+c^2\right)^{1.5} - \left(b^2+c^2\right)^{1.5}\right)\Bigg) \end{aligned} \tag{6}$$

Using the dimensions $10 \times 25 \times 0.75\,\mu$m^3, we find $N_L \approx 0.8762$, so that

$$B_{int} = \mu_0 H\left(1 - N_L\chi^L)\right) = \mu_0 H\left(1 - N_L\frac{\chi^{sph}}{1 - \chi^{sph}(N_{sph} - N_L)}\right) \approx 0.2023\,\mu_0 H \tag{7}$$

Hence, for the lamella, an applied magnetic field of 70 mT corresponds to $70 \times 0.2023 \approx 14.1\,$mT of internal field, which corresponds to $14.1/0.4 \approx 35.3\,$mT of applied magnetic field for the spherical

sample measured in SANS.

[1] H. Wilhelm, M. Baenitz, M. Schmidt, U. K. Rößler, A. A. Leonov, and A. N. Bogdanov, "Precursor Phenomena at the Magnetic Ordering of the Cubic Helimagnet FeGe," Phys. Rev. Lett. **107**, 127203 (2011).

[2] H. Wilhelm, A. O. Leonov, U. K. Rößler, P. Burger, F. Hardy, C. Meingast, M. E. Gruner, W. Schnelle, M. Schmidt, and M. Baenitz, "Scaling study and thermodynamic properties of the cubic helimagnet FeGe," Phys. Rev. B **94**, 144424 (2016).

[3] B Lebech, J Bernhard, and T Freltoft, "Magnetic structures of cubic FeGe studied by small-angle neutron scattering," Journal of Physics: Condensed Matter **1**, 6105–6122 (1989).

[4] X Z Yu, N Kanazawa, Y Onose, K Kimoto, W Z Zhang, S Ishiwata, Y Matsui, and Y Tokura, "Near room-temperature formation of a skyrmion crystal in thin-films of the helimagnet FeGe." Nature materials **10**, 106–109 (2011).

[5] E. Moskvin, S. Grigoriev, V. Dyadkin, H. Eckerlebe, M. Baenitz, M. Schmidt, and H. Wilhelm, "Complex Chiral Modulations in FeGe Close to Magnetic Ordering," Phys. Rev. Lett. **110**, 077207 (2013).

[6] A. Bauer, A. Neubauer, C. Franz, W. Münzer, M. Garst, and C. Pfleiderer, "Quantum phase transitions in single-crystal $Mn_{1-x}Fe_xSi$ and $Mn_{1-x}Co_xSi$: Crystal growth, magnetization, ac susceptibility, and specific heat," Physical Review B **82**, 064404 (2010).

[7] R. Ritz, in *Ph.D. Thesis, Superconductivity and non-Fermi liquid behavior on the border of itinerant ferromagnetism* (2010) pp. 113–115.

[8] Matthew J. Stolt, Zi-An Li, Brandon Phillips, Dongsheng Song, Nitish Mathur, Rafal E. Dunin-Borkowski, and Song Jin, "Selective Chemical Vapor Deposition Growth of Cubic FeGe Nanowires That Support Stabilized Magnetic Skyrmions," Nano Letters , acs.nanolett.6b04548 (2016).

[9] J. A. Osborn, "Demagnetizing Factors of the General Ellipsoid," Phys. Rev. **67**, 351–357 (1945).

[10] Amikam Aharoni, "Demagnetizing factors for rectangular ferromagnetic prisms," Journal of Applied Physics **83**, 3432–3434 (1998).
